# Efficiency analysis of commercial polymeric membranes for bone regeneration in rat cranial defects

**DOI:** 10.1590/acb380623

**Published:** 2023-03-06

**Authors:** Lana Karine Araújo, Mirrael de Sousa Lopes, Francisco Fábio Pereira de Souza, Marcelo Miranda de Melo, Anderson de Oliveira Paulo, Igor Iuco Castro-Silva

**Affiliations:** 1 Universidade Federal do Ceará – Postgraduate Program in Biotechnology – Sobral (CE), Brazil.; 2 Universidade Federal do Ceará – Dental School – Sobral (CE), Brazil.; 3 Universidade Federal do Ceará – Postgraduate Program in Biotechnology of Natural Resources – Fortaleza (CE), Brazil.; 4 Instituto de Educação Superior de Brasília – Dental School – Brasilia (DF), Brazil.

**Keywords:** Biocompatible Materials, Bone Regeneration, Guided Tissue Regeneration, Collagen, Polymers, Materials Testing

## Abstract

**Purpose:**

To evaluate the in vivo efficiency of commercial polymeric membranes for guided bone regeneration.

**Methods:**

Rat calvarial critical size defects was treated with LuminaCoat (LC), Surgitime PTFE (SP), GenDerm (GD), Pratix (PR), Techgraft (TG) or control (C-) and histomorphometric analysis determined the percentage of new bone, connective tissue and biomaterial at 1 or 3 months. Statistical analysis used ANOVA with Tukey’s post-test for means at same experimental time and the paired Student’s t test between the two periods, considering p < 0.05.

**Results:**

New bone at 1 month was higher for SP, TG and C-, at 3 months there were no differences, and between 1 and 3 months PR had greater increase growthing. Connective tissue at 1 month was higher for C-, at 3 months for PR, TG and C-, and between 1 and 3 months C- had sharp decline. Biomaterial at 1 month was higher for LC, in 3 months for SP and TG, and between 1 and 3 months, LC, GD and TG had more decreasing mean.

**Conclusions:**

SP had greater osteopromotive capacity and limitation of connective ingrowth, but did not exhibit degradation. PR and TG had favorable osteopromotion, LC less connective tissue and GD more accelerated biodegradation.

## Introduction

Severe bone losses caused by fractures and/or pathologies can generate functional or aesthetic changes, affecting the quality of life of affected patients^1^. Despite new technologies, their clinical treatment in implantology, periodontics and oral and maxillofacial surgery remains a challenge^2^. Bone regeneration occurs to a limited extent in large defects, requiring the use of osteopromotive biomaterials capable of enhancing this repair^3^. Guided bone regeneration (GBR) use membrane barriers associated with grafts in the region of the defect adjacent to teeth, post-extraction sockets or implants, in order to prevent soft tissue invasion and create a biologically suitable environment for osteogenesis and bone maturation^4^^,^ 5. Ideal characteristics for membranes include: biocompatibility, occlusiveness, osteopromotion^5^^,^ 6 and resorption to avoid second surgical time^7^^,^ 8.

Polymeric GBR membranes are the most used in the biomedical area^7^^,^ 9 ^,^ 10. Synthetic membranes include polytetrafluoroethylene (PTFE), resistant to degradation, bioinert, chemically stable, porous and flexible, and poly(lactic acid-co-glycolic acid) (PLGA), which has controlled degradation^11^^–^ 13. Natural membranes are usually of collagen, a biomimetic composition to favor the bone repair process^14^^–^ 17, with excellent biocompatibility, variable biodegradability^7^^,^ 18 and extraction sources, such as tendon, cortical bone or bovine pericardium^11^^,^ 19 ^,^ 20 or porcine submucosa^21^^,^ 22. Resorption time of polymeric membranes range from 4 weeks to 6 months, occuring gradually so that bone formation is possible^8^^,^ 23. Degradation control can be related to microporosities < 3 μm, which mantain nutritional diffusion without impairing the mechanical stability^24^^,^ 25, use of fibrillar matrix crosslinking techniques or its association with apatite^26^^,^ 27 or thicknesses from 0.1 to 1 mm to achieve desirable osteopromotive efficacy^28^, with greater densities delaying its total degradation^29^.

Despite of multiple options for GBR membranes in the dental market, there is no scientific consensus to indicate a gold standard that align simultaneously osteopromotive efficiency, physical barrier action against soft tissue ingrowth and balanced resorption during tissue repair, as desirable properties^30^. This study compared the performance of different commercial membranes in an experimental in vivo model of a critical size defect in rat calvaria, evaluating bone and connective tissue formation and residual biomaterials.

## Methods

### Ethical aspects

This study adopted the international principles of Substitution, Reduction and Refinement in Animal Research: Reporting of In vivo Experiments (3R-ARRIVE guide). The protocol was approved by the Ethics Committee on the Use of Animals of the Federal University of Ceará, Sobral, Brazil, under protocol number 06/2020.

### Commercial samples of GBR membranes

Five commercial products approved for clinical dental use in Brazil were selected as test groups^31^^–^ 35 and are described in Table 1. All GBR membranes had their commercial dimensions adapted to individual 10 mm^2^ square samples, necessary for the in vivo study. After customization, the materials were handled aseptically until the implantation procedure.

**Table 1:** Groups of commercial polymeric membranes for GBR used in this study.

Group	Product	Polymeric matrix	Manufacturing properties
LC	LuminaCoat (Criteria, Brazil), Batch: LC035/20, ANVISA n.: 80522420002	Natural bovine collagen type I	30 × 20 × 1 mm , resorbable after 4–6 weeks^31^
SP	Surgitime PTFE (Bionnovation, Brazil), Batch: 069922, ANVISAn.: 10392710009	Synthetic PTFE	30 × 20 × 0.25 mm,no resorbable^32^
GD	GenDerm (Baumer, Brazil), Batch: 4336456/004345315, ANVISAn.: 10345500069	Natural demineralized bovine cortical bone	20 × 20 × 0.15–0.20 mm, resorbable after 45 days^33^
PR	Pratix (Baumer, Brazil), Batch: 004344590, ANVISA n.: 10345500133	Synthetic PLGA	40 × 30 × 0.15–0.20 mm, resorbable after90–120 days^34^
TG	Techgraft (Baumer, Brazil), Batch: 004351444, ANVISA n.: 10345500141	Natural bovine acellular pericardium	20 × 20 × 0.15–0.25 mm, resorbable in 4–6 months^35^

### Implants in rat critical size bone defects

Sixty animals were distributed according to different experimental conditions (6 groups, 2 times, 5 animals each). The animals were given intramuscular anesthesia with 10% ketamine solution (Dopalen, Sespo Indústria e Comércio Ltda, Brazil) at a dose of 100mg/kg and xylazine 2% (Anasedan, Sespo Indústria e Comércio Ltda, Brazil) at a dose of 10 mg/kg. Then, there was trichotomy of the upper part of the head and antisepsis with 0.5% aqueous chlorhexidine. A semilunar incision was made followed by a mucoperiosteal flap, reflected with Molt’s periosteal elevator, exposing the cortical bone in the frontoparietal region. A single, 8-mm diameter circular defect of critical size was created in each animal using a surgical trephine drill (Sistema de Implantes Nacionais, Brazil) attached to a contra-angle with 20:1 rotation reduction (Dentscler, Brazil) and a surgical micromotor (VK Driller Equipamentos Elétricos Ltda., Brazil) under irrigation conditions with cold and sterile 0.9% saline solution during the procedure. The osteotomized fragment was gently removed using an Ochsenbein #1 chisel. The test groups had the bone defect filled by one of the materials (G1, G2, G3, G4 or G5). As a negative control (C-), it was adopted a natural filling with blood clot after the bone defect. The operated regions had simple sutures with 4.0 mononylon thread. Subcutaneous anti-inflammatory/analgesic medication Meloxicam (2 mg/kg, Ourofino, Brazil) was applied every 12 h for 2 days. At 1 and 3 months after the surgeries, the animals were euthanized by an overdose of anesthetic solution and an immediate excisional necropsy of the area compatible with each surgery was performed.

### Histotechnic, histological and histomorphometric analysis

The samples were fixed in 10% buffered formalin solution (v/v), pH 7.0, for 48 h. After fixation, all necropsies were decalcified with rapid acid decalcifying solution (Allkimia, Brazil) for 4 days, washed in running water for 1 h, cleaved with a razor in the center of the bone defect, dehydrated in increasing baths from 70 to 100% of ethanol, cleared in xylol baths, impregnated and embedded in paraffin, evidencing the central region of the bone defect. The paraffin blocks were microtomized in 4μm sections and stained in hematoxylin-eosin (HE).

Biological phenomena were analyzed in qualitative and quantitative perspectives. Five images of each sample were captured in adjacent, non-overlapping fields using the Cybershot DSC-W300 Super Steady Shoot camera (Sony, Japan) coupled with the FWL-1000 optical microscope (Feldman Wild Leitz, Brazil) using a 10x objective lens, 10x ocular lens and 4× digital zoom, making a final magnification of 400×. For qualitative analysis, slides from each test and control group were selected and morphologically described to represent the observed events. The following biological criteria were evaluated on the edge-to-edge extension of the bone defect, covering its entire diameter: newly formed bone, connective tissue and implanted biomaterial.

**Figure 1:**
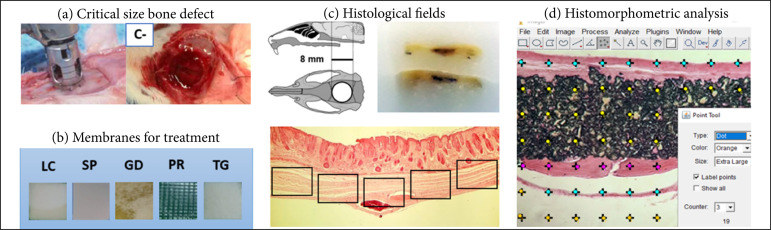
Steps of the in vivo procedure. **(a)** Surgical creation of critical size bone efect (CSD) without coating in the control (C-); **(b)** Membranes for treatment: LuminaCoat (LC), Surgitime PTFE (SP), GenDerm (GD), Pratix (PR) or TechGraft (TG); **(c)** After cleavage of the samples and processing in paraffin, 5 fields were photocaptured per histological slide along the CSD; **(d)** Histomorphometric analysis by points using ImageJ software, with different biological criteria distinguished by colors.

Quantitative histomorphometric analysis was performed using the ImageJ 1.52a version software (National Institutes of Health, USA), calibrated in micrometers/pixel. The biological criteria mentioned above were counted using a grid of 130 points superimposed on each photomicrograph and from the absolute number of points obtained, the percentage volume density (%i) of each parameter was determined according to the Eq. 1:


$$\%i=\left(\frac{pi}{p}\right)100\%$$ (1)

where *p*_i_ represents the number of poins in each parameter and *P* the total number of points. Figure 1 summarizes the in vivo characterization procedures of this study.

Data for each parameter were tabulated in Excel software (Microsoft Office, USA), expressed graphically as mean±standard deviation and statistically analyzed using the Prism 7.0 software (GraphPad, USA) for comparisons of groups and experimental times. Analysis of variance (ANOVA) with Tukey’s post-test was applied to analyze the normal/parametric distribution of the means of each parameter between the five experimental groups and the control at each experimental time. Paired Student’s t test was applied to analyze the normal/parametric distribution of the means of each parameter between the five experimental groups and the control at each experimental time, as dependent samples. They were considered confidence level of 95% and significant differences if p < 0.05.

## Results

**Figure 2:**
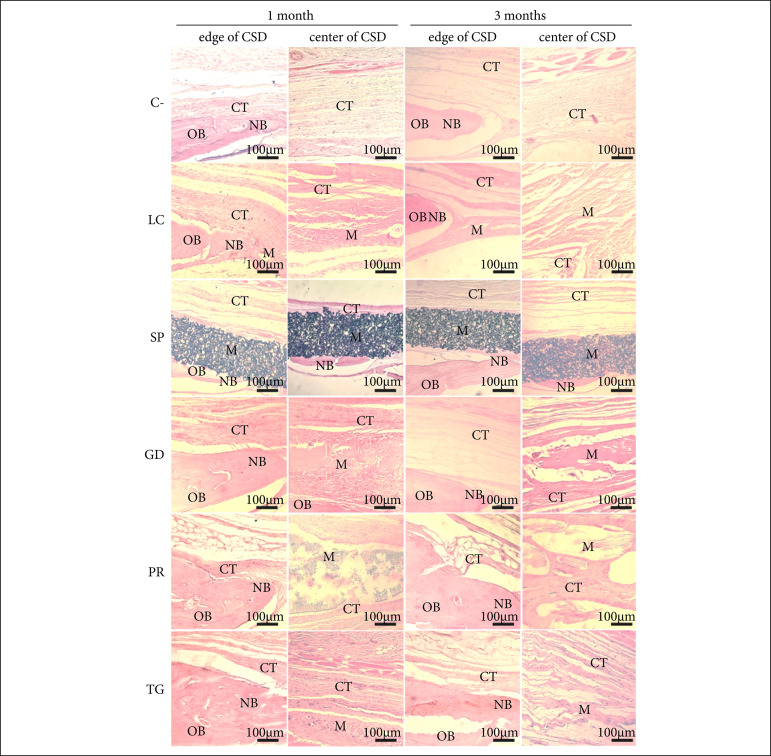
Histological analysis in critical size bone defects (CSD) in rat calvaria for the different experimental groups at one and three months. Qualitative data of control (C-), LuminaCoat (LC), Surgitime PTFE (SP), GenDerm (GD), Pratix (PR) and TechGraft (TG). All groups exhibited the most prominent presence of new bone (NB) at the edge of the CSD, adjacent to native old bone (OB), while at the center of the CSD there were varying amounts of connective tissue (CT) and/or residual membrane **(M)**.

The histological analysis showed that all treatments and C- showed a small amount of newly formed bone closer to the edges of the bone defects, greater than the islets of bone in its most central region, with a progressive increase in centripetal osteogenesis between 1 and 3 months. The connective tissue was more abundant in C- compared to the other groups, evolving from a loose extracellular matrix in 1 month to a more fibrous tissue in 3 months. It was possible to observe the presence of material in up to 3 months in all groups except for C-, with no noticeable degradation for SP and PR, while LC and GD showed evident degradation of the material between 1 and 3 months (Fig. 2).

**Figure 3:**
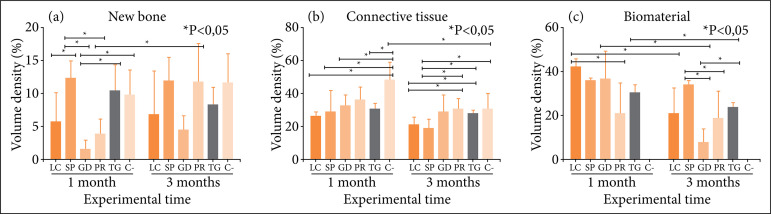
Histomorphometric analysis of volume density of **(a)** new bone, **(b)** connective tissue, and **(c)** biomaterial in critical size bone defects in rat calvaria for the different experimental groups at one and three months. Percentage data of control (C-), LuminaCoat (LC), Surgitime PTFE (SP), GenDerm (GD), Pratix (PR) and TechGraft (TG).

The histomorphometric analysis showed significant differences for the percentage of new bone at 1 month between the groups, with the mean of SP (12.26 ± 2.83%) being higher than the means of LC (5.64 ± 4.54%), PR (3.96 ± 2.19%) and GD (1.44 ± 1.31%) and the means of TG (10.38 ± 3.95%) and C- (9.81 ± 3.68%) being higher than the average of GD (1.44 ±1.31%). In the experimental period of 3 months, there was no significant difference between the groups (p = 0.074). In the evaluation between the experimental times, there was a significant difference for the mean of PR, increasing from 3.96 ± 2.19% in 1 month to 11.66 ± 5.94% in 3 months (Fig. 3a).

There were significant differences for the percentage of connective tissue at 1 month, with the mean of C- (48.43 ± 10.54%) surpassing the means of GD (32.51 ± 6.49%), TG (30.85 ± 3.29%), SP (28.46 ± 12.71%) and LC (25.79 ± 2.87%). In 3 months, the means of PR (31.01 ± 5.96%) and TG (27.65 ± 2.27%) exceeded those of LC (20.65 ± 4.88%) and SP (18.85 ± 5.75%), as well as C- (30.74 ± 9.15%) was higher than SP (18.85 ± 5.75%). In the evaluation between the experimental times, there was a significant difference for C-, decreasing from 48.43 ± 10.54% in 1 month to 30.74 ± 9.15% in 3 months (Fig. 3b).

There were significant differences for the percentage of biomaterial at 1 month, with the mean of LC (42.09 ± 4.28%) surpassing the mean of PR (21.25 ± 13.84%). In the period of 3 months, the average of SP (34.64 ± 1.42%) surpassed the averages of PR (18.80 ± 12.21%) and GD (7.70 ± 6.35%), while the mean of TG (23.57 ± 2.27%) was higher than the mean of GD. In the evaluation between the experimental times, there was a significant difference between 1 and 3 months, for LC (from 42.09 ± 4.28% to 20.91 ± 11.88%), GD (from 36, 37 ± 13.01% to 7.70 ± 6.35%) and TG (from 30.28 ± 3.84% to 23.57 ± 2.27%), which proves the presence of biodegradation in these groups (Fig. 3c).

Considering the results achieved over the 3 months of the experiment and the individual requirements for choosing an ideal regenerative membrane, the decreasing order of efficiency in terms of osteopromotive capacity would be: SP > PR > TG > LC > GD. As for the smallest tendency to formation of connective tissue, the decreasing order of efficiency would be: SP > LC > TG > GD > PR. Finally, regarding the presence of biodegradation, from the most accelerated modality to the non-resorption modality, the decreasing order of efficiency would be: GD > PR > LC > TG > SP. SP had greater osteopromotive capacity and limitation of connective tissue ingrowth, but did not exhibit degradation. PR and TG had favorable osteopromotion, LC less connective tissue and GD more accelerated biodegradation.

## Discussion

Semiautomated histomorphometric analysis with manual point counting in software along the length of the critical defect is used for initial estimation of inflammation and neovascularization between 7 and 15 days post-surgery^36^^–^ 38 or for its main objective of analyzing the percentage of bone formation and maturity, secondarily evaluating connective tissue and residual biomaterial, between 1 and 3 months after surgery^4^^,^ 17 ^,^ 39. Despite the automated counting in software by delimited area in pixels converted into mm^2^ is a fast resource and used with great popularity in thematic research^19^^,^ 27 ^,^ 36 ^–^ 38 ^,^ 40 ^–^ 44, the semiautomated counting values the histopathological diagnosis, allowing the optical distinction of new bone from old or native bone at the edges of the bone defect, as well as fragments of collagenous biomaterial against connective tissue fibers, making the calculation of these parameters more accurate^4^^,^ 17 ^,^ 39. Such scientific evidence makes this research robust, unbiased and accurate for the analysis of osteopromotive membranes for GBR.

Critical defects in rat calvaria in control group without the use of membrane generally cause small percentages of newly formed bone, ranging from 0.4%^43^ to 4% at 1 month^4^^,^ 17 ^,^ 27 and from 0.9%^40^ to 5% in 3 months^4^^,^ 17 or reaching up to 20%, considering the average of central regions with up to 2%, intermediate up to 8% and peripheral regions up to 40%^39^. The amount of connective tissue remains constant between 1 and 3 months, with about 23%^4^ or can reach up to 40% in the aforementioned periods^17^.

In the experimental rat skull model, membranes for GBR alone can achieve different osteopromotive profiles. In the period of 1 and 2 months, the ratio of area of newly formed bone compared to control group can be two to ten times for Bio-Gide with collagens I and III from swine dermis^19^^,^ 36 ^–^ 38 or Jason with collagen III from porcine pericardium^38^^,^ 42, equal to five times for bovine GenDermFlex^19^, two to four times for Collprotect with collagen from porcine dermis^38^ or Super Fixorb with polylactic acid and hydroxyapatite (60:40)^40^ and two to three times for bovine GenDerm^19^^,^ 42, and for synthetic membrane with policaprolactone and 5% hydroxyapatite^37^. There are cases such as the Biomend with collagen I that presents formation of new bone similar to control group^40^, the bacterial cellulose membrane bellow of the control group in 1 month and above until five times its area in 2 months^36^ and the PLGA membrane, with no differences compared to control group at 2 months^45^, showing that the performance of these implantable devices can vary greatly, depending on the type and duration of treatment.

Collagen membrane alone after 1 month of implantation in a bone defect can generate 8% of new bone and 0.3% of remnants, and when associated with bone graft, it reaches 15% of new bone and 0.03% of remnants^27^. The combination of resorbable collagen membrane Cola-D and xenografts Bio-Oss (bovine; granulometry: 0.25–1 mm) or Bone-XP (porcine; granulometry: 0.2–1 mm) can generate new bone, 5.83% and 9.08% at 1 month and 21.68% and 25.22% at 2 months, respectively^44^. Another study with Bio-Oss (granulometry: 0.5–1 mm) coated with BioGide in 1 or 2 layers showed that the single coating generated in 1 and 2 months, respectively, more bone (22.7% and 37%) than the double (17.3% and 24.5%) or the graft without membrane (11.5% and 16.8%), although the amount of residual material was slightly higher in the double (30.2% and 25.5%) than in simple (32.5% and 28.5%) or with graft without membrane (15.3% and 9.4%)^43^. In rat tibia defects, membrane GenDerm alone could favor new bone formation by 25.3% at 1 month and 32.2% at 3 months, and when associated with organic bovine graft GenOx, it increased bone formation to 45.5% and 52.4%, respectively^46^.

Regarding the formation of connective tissue, there was no variation between the membranes studied. However, the literature admits some microscopic distinctions in fibrogenesis, according to the type and duration of treatment. Metallic nonresorbable biomaterials (e.g., titanium) have more discrete fibrogenesis because they are bioinert^47^. Natural resorbable polymers (e.g., collagen) exhibit greater peripheral and internal cellularity, mild to moderate chronic inflammation (lymphocytes, macrophages and giant cells) and fibroblast proliferation, in addition to mild to moderate production of connective matrix in the spaces left by the degrading implant up to 60 days^11^^,^ 46 ^–^ 51. Natural composite membranes with collagen and apatite also exhibit fibrogenesis similar to collagen biopolymers^17^^,^ 50 ^,^ 52. On the other hand, synthetic polymers present connective tissue formation dependent on the degradability pattern, in a lower fibrogenesis scale in non-resorbable biomaterials (e.g., PTFE)^11^^,^ 12 ^,^ 24 ^,^ 47 ^,^ 53 and higher in resorbable ones (e.g., PLGA)^13^^,^ 24 ^,^ 45 ^,^ 47 ^,^ 54 ^,^ 55. None of the experimental groups had a foreign body granuloma (nonimmunogenic) with a fibrous capsule, which is considered an unwanted implant rejection response^17^^,^ 18 ^,^ 47.

Regarding the biodegradability of membranes in rats, Lumina-Coat confirmed the stability time of 4 to 6 weeks^11^^,^ 31, falling short of the Lumina-Coat Double Time, stable up to 8 weeks^50^^,^ 56. Surgitime PTFE did not show resorption during the experiment, as expected for the synthetic polymer PTFE^11^^,^ 12 ^,^ 24 ^,^ 32 ^,^ 53. Despite GenDerm being well organized, with high tensile strength and less deformation compared to Lumina-Coat and Surgidry Dental F, internal cracks explain structural fragility and greater subcutaneous degradation, predicted for up to 45 days^11^^,^ 33. GenDerm fragments disappear after 30 days in tibial defects^24^^,^ 46, which resembles another demineralized bone cortical membrane, intact in the subcutaneous tissue at 15 days, degraded in 30 days and absent in 60 days^20^ and differs from BioGide, with subcutaneous residues present up to 63 days^48^ or mild degradation in swine mandibular bone defects up to 12 weeks and disappearing at 27 weeks^22^. The resorption time predicted between 90 and 120 days of Pratix^34^ becomes plausible, as it practically did not change, according to the pattern observed in subcutaneous tissue in 254 or 3 months^55^ and already expected for PLGA, with high tensile strength^57^ and slower degradation^13^^,^ 24, demonstrated subcutaneously from 4 to 26 weeks^45^. Techgraft outperformed subcutaneous bovine pericardium membranes, intact in 15 days and absent between 30 and 60 days^20^^,^ 49. The biodegradation between 4 and 6 months suggested for Techgraft^35^ converges with the same time observed with Jason in subcutaneous tissue^38^^,^ 42 and in swine mandibular defects, slightly degraded up to 12 weeks and absent at 27 weeks^22^.

Preclinical studies look for membranes with adequate biocompatibility for application to GBR^5^^,^ 51. In general, Bio-Gide and Jason are membranes that present a good pattern of non-irritation, considering the inflammatory and repair response^51^. Regarding the tissue dynamics involved, the lower presence of inflammatory cells and twice the number of blood vessels in the first fifteen days after implant of Bio-Gide can explain its better performance in comparison to bacterial cellulose membranes, polycaprolactone with 5% hydroxyapatite, Jason and Collprotect^36^^–^ 38. Bio-Gide also outnumbered blood vessels up to 21 days in relation to bovine membrane Lyostypt^48^. This behavior could be associated with the pro-angiogenic effect of collagens I and III, which would favor osteogenesis^16^^,^ 30.

Regarding the osseodifferentiation process, the higher immunoexpression of osteocalcin and lower of osteopontin between 1 and 2 months of implantation in the calvaria could be interpreted as favorable bioindicators of greater bone maturation for membrane Bio-Gide, both when compared to cellulosic membrane^36^ and to collagen swine membranes Jason and Collprotect^38^. Similar results were found when comparing the porcine membrane Bio-Gide with the bovine membranes GenDerm and GenDermFlex, with higher expression of ostecalcin with porcine origin and of osteopontin with bovine origin at 1 and 2 months of implantation, showing a potential correlation between animal source and performance of GBR membranes^19^^,^ 42. Fibrous organization of natural collagenic matrices from different sources may explain differences in osteopromotion and degradation time^58^^,^ 59.

Porosity is a very sensitive characteristic of GBR membranes, as nanometric porosities (pores of 0.004 μm) into collagen membrane are associated with the same amount of newly formed bone as the control group^40^. On the other hand, a greater porosity reduces the osteopromotive capacity, as in the case of a titanium membrane without pores, which reached a greater area of neoformed bone compared to both anodized membranes with pores of 0.4 and 1.5 mm, in the ratio 1.6:1, as well as increased immunoexpression of calcein for up to 7 weeks^41^. Surgitime PTFE features more interconnected synthetic polymer fibers, providing less permeability and greater mechanical resistance compared to GenDerm and Lumina-Coat, natural polymers with a heterogeneous distribution of collagen fibers, which gives them highly porous surfaces with varied diameters^42^, justifying the present results of biodegradation in natural membranes.

As for thickness, most commercial membranes tested are close to the range of 0.10 to 0.25 mm, most commonly reported in polymeric materials for GBR^11^^,^ 18 ^,^ 24 ^,^ 28 ^,^ 38 ^,^ 40 ^,^ 45 ^,^ 46 ^,^ 57. Membranes beyond this range include Collprotect at 0.4 mm thickness^38^, Lumina-Coat at 1 mm^11^ or Lumina-Coat Double Time at 2 mm^50^^,^ 56. The thicker membrane design attempts to mechanically ensure its tissue barrier function, but contributes to a slower and more persistent inflammatory response, which can increase the pattern of irritation to the biomaterial^16^^,^ 17 ^,^ 50 ^,^ 56. When comparing Lumina-Coat and Lumina-Coat Double Time, the degradation time doubles in the same proportion as its thickness, but it becomes more rigid and less attractive to surgical manipulation in small intraoral bone defects^11^^,^ 50 ^,^ 56. In composite membranes of PLGA, HA and βTCP, the thickness of 0.2 mm maintained the integrity of the material for up to 30 days, while thicknesses of 0.5 and 0.7 mm reached 90 days^55^. This logic does not always work, as the membrane Jason is half as thick and three times less dense than Bio-Gide, but both have similar biodegradation times^22^^,^ 38. To overcome these limitations, microstructural reinforcement has been more used, with chemical processes involving crosslinking, in order to keep the membrane thinning, its good adaptability to the bone defect and, at the same time, cohesion for a longer time to favor osteopromotion^16^^,^ 17 ^,^ 30.

The detailed description of a biomaterial is essential and should avoid scenarios with technical unconformities, as observed in a study with commercial grafts in Brazil, where erroneous information in the product package insert on physical-chemical characteristics was revealed in an independent test^57^. The explicit biological data regarding osteogenesis, fibrogenesis and degradation of the tested regenerative membranes can contribute to decision making, good clinical planning and predictability of results in GBR.

## Conclusion

The commercial membranes LuminaCoat, Surgitime PTFE, GenDerm, Pratix and Techgraft showed heterogeneous behavior in rat calvarial defects. Although Surgitime PTFE had greater osteopromotive capacity and limitation of connective tissue invagination, the biomaterial did not exhibit degradation. Pratix and Techgraft had favorable osteopromotion, LuminaCoat less connective tissue and GenDerm more accelerated biodegradation.

However, the most effective GBR membrane that simultaneously contemplates all the studied criteria remains undefined. The difficulty in meeting the set of specificities for choosing an ideal regenerative membrane raises future studies about the intrinsic factors of each biomaterial.
